# Stage-Stratified Incidence Rates of Colorectal Adenocarcinoma Among Patients Aged 46 to 49 in the United States

**DOI:** 10.1001/jamanetworkopen.2024.1848

**Published:** 2024-03-15

**Authors:** Eric M. Montminy, Meijiao Zhou, Jerome C. Edelson, Jordan J. Karlitz

**Affiliations:** 1 Division of Gastroenterology, John H. Stroger Jr Hospital of Cook County, Chicago, Illinois; 2 Southborough, Massachusetts; 3 Division of Gastroenterology, Brooke Army Medical Center, Fort Sam Houston, Texas; 4 Division of Gastroenterology, University of Colorado School of Medicine, Aurora

## Abstract

This cross-sectional study uses Surveillance, Epidemiology, and End Results registry data to analyze colorectal adenocarcinoma staging incidence of patients aged 46 to 49 years from 2000 to 2020.

## Introduction

Colorectal adenocarcinoma incidence rates (IR) are rising in patients less than 50 years of age.^1^ These adenocarcinomas predominantly display more advanced and nonlocalized stages (ie, regional and distant), and many patients in their late 40s may harbor preclinical advanced lesions that are ultimately diagnosed with screening at age 50.^2^ The United States Preventive Services Task Force recommended average-risk screening begin at 45 years of age.^1^ However, the American College of Physicians (ACP) recently recommended screening initiation remain at 50 years of age because of limited clinical trial evidence of screening benefits.^3^ Conflicting guidelines can cause patient confusion (such as recent conflicting breast cancer screening recommendations) for those between ages 45 and 50 years and increase delays in screening past 45 years of age and into ages 46 to 49 years.^4^ Because people aged 46 to 49 years are between the 2 recommendations, this group is at increased risk for confusion and warrants focused incidence analysis for further screening guidance. To our knowledge, colorectal adenocarcinoma incidence rate analysis of those aged 46 to 49 has not been performed. Staging analysis in this age group is important as an increasing burden of advanced staged disease would provide further evidence for earlier screening initiation. Here, we provide analysis of colorectal adenocarcinoma staging incidence of patients aged 46 to 49 years from 2000 to 2020 using Surveillance, Epidemiology, and End Results (SEER) registry data.

## Methods

This cross-sectional study was deemed exempt and granted a waiver of informed consent by the Cook County Hospital institutional review board due to using deidentified patient data from a national registry. The STROBE reporting guideline was followed.

A cross-sectional analysis of 2000 to 2020 SEER 17 annual age-adjusted IRs per 100 000 of patients aged 46 to 49 years was performed using SEER*Stat. IRs were collected for colorectal site, only adenocarcinoma histology, and all races and sexes combined. Race and sex data were collected from SEER. IRs were stratified by stage: localized, regional, and distant. High-grade dysplasia is not recorded by SEER, therefore in situ cancers were excluded. Joinpoint Regression Program version 5.0.2 (SEER*Stat) quantified yearly annual percentage change (APC) by stage. Two-sided *P* < .05 was considered statistically significant. Statistical analysis was performed in July 2023.

To determine statistically significant differences in absolute adenocarcinoma IRs within subgroups, adjusted rate ratios (ARRs) were assessed. These were calculated for each calendar year and stratified by staging subgroups. Significant differences between subgroups were present when ARR 95% CIs did not cross 1.

## Results

From 2000 to 2020, 26 887 colorectal adenocarcinomas were diagnosed in adults aged 46 to 49 years. Of these adenocarcinomas, 14 329 (54.5%) were in men and 11 960 (45.5%) were in women; 2607 (9.9%) were in Asian patients, 3565 (13.6%) were in Black patients, and 19 647 (74.7%) were in White patients. As of 2020, localized adenocarcinoma IR decreased to 7.7 of 100 000, regional adenocarcinoma IR increased to 13.4 of 100 000, and distant adenocarcinoma IR increased to 9.0 of 100 000. Regional adenocarcinoma IR remained the highest of all stages from 2000 to 2020. From 2014 to 2020, distant IRs became similar to localized IRs (ie, adjusted rates ratios were no longer significant with overlapping 95% CIs), except in 2017 when distant IR was significantly higher than localized (Figure).

**Figure.**
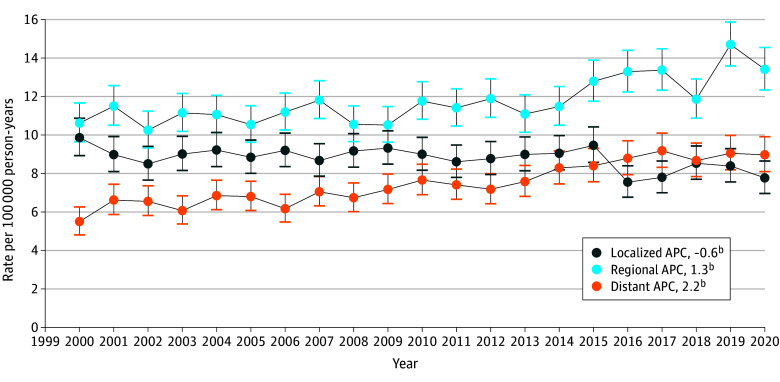
Colorectal Adenocarcinoma Staging Incidence Rates per 100 000 Person-Years and Annual Percentage Change (APC) in People Aged 46 to 49 Years From 2000 to 2020^a^ ^a^Data obtained from Surveillance, Epidemiology, and End Results 17 registry data. ^b^Indicates statistical significance to *P* < .05.

Regarding APCs, distant adenocarcinoma IRs increased faster than other stages (APC, 2.2 [95% CI, 1.8 to 2.6]). Regional IRs significantly increased (APC, 1.3 [95% CI, 0.8 to 1.7]). Only localized IRs decreased (APC, −0.6 [95% CI, −1.0 to −0.2]).

## Discussion

This cross-sectional study found that distant stage colorectal adenocarcinoma, as well as regional stage disease, has been increasing in recent years in individuals aged 46 to 49 in the setting of now conflicting national screening guidelines. For context, CRC absolute regional IRs in those aged 46 to 49 years are similar with total pancreatic cancer IRs in all ages and all stages combined (13.2 of 100 000) over similar years. When including distant CRC adenocarcinoma IRs with CRC regional IRs, CRC IRs are double pancreatic cancer IRs of all stages combined.^5^ Importantly, a recent study demonstrated a 46% increase in CRC IRs from the ages of 49 to 50, highlighting that there is a large preclinical CRC burden in those in their 40s that was not detected until screening initiation at age 50. Thus, in our current analysis, which did not span the calendar years when age 45 screening was routinely recommended, CRC absolute IRs may be an underestimate.^2^ Our results demonstrate that adults aged 46 to 49 years, who are between now conflicting guidelines on whether to start screening at age 45 or 50, have an increasing burden of more advanced stage CRC and thus may be at increased risk if screening is not initiated at age 45. Advanced adenocarcinoma is associated with considerable morbidity and mortality and often more invasive and costlier therapy.

Our findings are consistent with a recent colorectal adenocarcinoma analysis that measured stage IRs in 10-year age blocks under age 50, which demonstrated shifts toward distant and regional adenocarcinoma.^6^ Limitations include our cross-sectional design and not addressing the ACP need for clinical trial data. However, our results provide a unique foundation among those aged 46 to 49 years from validated databases.

## Notes

Supplement.
Data Sharing Statement
