# Supplementary material for: Stage-Stratified Incidence Rates of Colorectal Adenocarcinoma Among Patients Aged 46 to 49 in the United States
Source: JAMA Netw Open. 2024 Mar 15;7(3):e241848. doi: 10.1001/jamanetworkopen.2024.1848 (PMC10943413; doi:10.1001/jamanetworkopen.2024.1848)
Supplement: Supplement. — Data Sharing Statement [file jamanetwopen-e241848-s001.pdf]

Data Sharing Statement

Montminy. Rates of Localized, Regional, and Distant Colorectal Adenocarcinoma Among Patients Aged 46 to 49 Years. *JAMA Netw Open*. Published March 15, 2024. doi:10.1001/jamanetworkopen.2024.1848

Data
Data available: Yes
Data types: Other (please specify)
Additional Information: SEER 17 data can be provided upon request
How to access data: SEER 17 data can be provided upon request
When available: With publication

Supporting Documents
Document types: Other (please specify)
Additional Information: SEER 17 data can be provided upon request
How to access documents: SEER 17 data can be provided upon request
When available: With publication

Additional Information
Who can access the data: Anyone who requests
Types of analyses: SEER 17 incidence data
Mechanisms of data availability: Upon request to the primary author.
Any additional restrictions: N/a
